# Salsolinol and ethanol-derived excitation of dopamine mesolimbic neurons: new insights

**DOI:** 10.3389/fnbeh.2013.00074

**Published:** 2013-06-25

**Authors:** Ana Polache, Luis Granero

**Affiliations:** Departament de Farmàcia i Tecnología Farmacéutica, Universitat de ValènciaBurjassot, Spain

Evidence supporting the essential role of brain-derived ethanol metabolites in the excitation of dopamine (DA) midbrain neurons has multiplied in the last 10–15 years. The pioneer and influential behavioral studies by CM Aragon and colleagues (see Correa et al., 2012 for a complete review) and more recent data (Sánchez-Catalán et al., 2009; Martí-Prats et al., 2010, 2013) have repeatedly demonstrated the crucial role displayed by acetaldehyde (ACD) in the locomotor and other behavioral responses elicited by ethanol. Although these experiments mainly used an indirect measure (exploratory locomotion) as an index of the excitation of DA neurons in the ventral tegmental area (VTA), results strongly suggested that the manipulations of ethanol brain metabolism determine the response (activation or not) of the DA neurons in the mesolimbic system after ethanol administration.

At the beginning of the past decade, a remarkable leap in the study of the ACD involvement in the ethanol-derived activation of VTA DA neurons was done. Several electrophysiological studies (Foddai et al., 2004; Melis et al., 2007) directly demonstrated that VTA DA neurons are not excited after ethanol administration if ACD production is inhibited. The strength of the provided evidence was very high because, unlike behavioral studies, a direct measure of the neuronal activity after administration of the drug was registered.

These direct and indirect findings confirmed the involvement of ACD in the excitation of the mesolimbic system. But, in spite of their relevance, an important question relative to ACD-derived excitation of the VTA DA neurons remains unresolved. If ACD is the responsible for the excitation of the DA mesolimbic system after ethanol administration, how ACD excites VTA DA neurons? In other words, what mechanism does ACD use to activate DA neurons? As occurs with ethanol, ACD has not any subset of specific receptors on nerve, glial, or other cells in the brain. Moreover, ACD, as other highly toxic aldehydes, is evanescent and reacts instantaneously with other compounds to form new products. Notably, biogenic amines are among the compounds that can react with ACD producing the so-called tetrahydroisoquinolines. The mesolimbic system is particularly enriched with DA, so in this brain region ACD locally formed after ethanol administration can react with DA forming salsolinol (Sal; 1-methyl-6,7-dihidroxy-1,2,3,4-tetrahydroisoquinoline) (Collins and Bigdeli, 1975; Nagatsu, 1997). In fact, in most cases, the results reported show that chronic ethanol treatment produces an increase of Sal levels in different brain areas, such as the striatum, hypothalamus and limbic regions. Moreover, it is also evidenced that the type of treatment i.e., the pattern of ethanol intake, determines the magnitude of enhancement of Sal in brain (see Hipólito et al., 2012 for review). So the question now is: Could Sal be the responsible for the VTA DA neuronal excitation after ethanol administration? In this issue of *Frontiers of Behavioral Neuroscience*, Xie and Ye review direct electrophysiological evidences supporting the role of Sal in the VTA DA neuronal activation after ethanol administration.

As occurred with the evidence supporting the role of ACD in ethanol effects, data initially provided by the scientific community on the involvement of Sal on the ethanol-derived VTA DA excitation were indirect. So, behavioral studies published in the last few years, showed that Sal directly administered into the posterior VTA is able to induce motor activation through a mechanism dependent on mu-opioid receptors (MORs) (Hipólito et al., 2010). Moreover, Sal is also able to induce motor sensitization and CPP (Hipólito et al., 2011), two behavioral responses closely related with activation of the DA mesolimbic system. Importantly, Sal is also self-administered into the posterior VTA (Rodd et al., 2008). A more direct proof supporting the ability of Sal to activate VTA DA neurons was the demonstration that microinjections of Sal into the posterior VTA increase DA levels in NAc shell (Hipólito et al., 2011; Deehan et al., 2013). Nonetheless, the most direct demonstration derives from recent studies using electrophyisiological recordings. To adequately appreciate the relevance of these new findings reviewed by Xie and Ye, it is important to remember that previous electrophysiological studies reported by this group in the past decade demonstrated that ethanol indirectly excites (disinhibits) VTA DA neurons (Xiao et al., 2007). Concretely, according to their data, ethanol inhibits, through a mechanism dependent on MORs, the neuronal activity of local GABA neurons which tonically inhibit the activity of VTA DA neurons (Johnson and North, 1992). Could Sal be the responsible for these exciting findings? Using whole-cell patch-clamp recordings to examine the effects of Sal on VTA DA neurons in acute brain slices, Ye and collaborators demonstrate that Sal stimulates DA neurons partly by reducing inhibitory GABAergic transmission through a mechanism dependent on MORs. Xie and Ye also review additional aspects on the mechanism of action of Sal, such as the ability of Sal to enhance presynaptic glutamatergic transmission onto VTA DA neurons via activation of DA type 1 receptors in the glutamatergic terminal. These and other novel aspects of the mechanism of action of Sal could be crucial to finally understand how ethanol interacts with DA mesolimbic system.

Collectively, the data reviewed suggest that Sal could be the responsible for the activation of the VTA DA neurons after ethanol administration. However, to definitively link Sal to ethanol-derived excitation of DA mesolimbic system after ethanol consumption, it is compulsory resolving, at least, two important interrogations. First, it is crucial to demonstrate the existence of an increase of VTA Sal levels after acute local or systemic ethanol administration. Second, it would be also decisive to establish a clear correlation between changes in VTA Sal levels and excitation of VTA DA neurons after ethanol administration. Future experiments on these crucial issues may be pivotal to unambiguously probe the involvement of Sal in the excitation of DA mesolimbic neurons after ethanol administration.
